# Electrodermal Activity, Behavior, and Context in Autistic People with High Support Needs: A Retrospective Ecological Study

**DOI:** 10.3390/healthcare14182990

**Published:** 2026-09-13

**Authors:** Marilia Baquerizo-Sedano, Miguel Lancho Pedrazo, María Merino Martínez, Álvaro Taype-Rondan, José Luis Cuesta Gómez, María Consuelo Sáiz-Manzanares

**Affiliations:** 1 Faculty of Health Sciences, University of Burgos, 09001 Burgos, Spain; mbs0077@alu.ubu.es (M.B.-S.); maria.merino@autismoburgos.org (M.M.M.); mcsmanzanares@ubu.es (M.C.S.-M.); 2 Graduate School, Universidad Continental, Huancayo 12001, Peru; 3 Autismo Burgos, 09001 Burgos, Spain; 4 Research Unit for the Generation and Synthesis of Health Evidence, Office of the Vice-President for Research, Universidad San Ignacio de Loyola, Lima 15024, Peru; 5 EviSalud—Health Evidence, Lima 15001, Peru; 6 Faculty of Education, University of Burgos, 09001 Burgos, Spain

**Keywords:** autism, high support needs, electrodermal activity, autonomic arousal, ecological momentary assessment, wearable sensors, routine-care data, contextual adjustment

## Abstract

**Highlights:**

**What are the main findings?**

- EDA patterns in autistic people with high support needs showed substantial within- and between-person variability in everyday settings.
- Elevated EDA did not, by itself, indicate distress; interpretation required consideration alongside behavioral and contextual records.

**What are the implications of the main findings?**

- Wearable monitoring in autism should use multimodal interpretation rather than treating EDA as a direct marker of stress.
- Individualized contextual adjustments may generate clinically useful hypotheses; however, prospective designs are required before causal conclusions can be drawn.

**Abstract:**

**Background/Objectives**: Wearable biometric sensors may help characterize autonomic arousal in everyday settings among autistic people with high support needs, a population underrepresented in naturalistic research. This study aimed to describe patterns of autonomic arousal measured using electrodermal activity (EDA) and to explore their relationship with behavioral and contextual records compatible with distress or maladaptive stress. **Methods**: We retrospectively analyzed routine-care data from 57 autistic people with high support needs attending two specialized services. EDA was recorded for up to 10 days and integrated with behavioral and contextual records collected through a mobile application and stored in ABmonitor. Phase 1 retrospectively analyzed these data using principles derived from Ecological Momentary Assessment. Phase 2 descriptively compared pre- and post-adjustment EDAmax values in five cases with documented contextual adjustments, drawing on principles of Ecological Momentary Intervention, without causal inference. **Results**: In Phase 1, 19.3% of participants remained within the same arousal category across days, whereas 80.7% showed variable levels. Overall, 22 of 57 participants (38.6%) showed high autonomic arousal on at least one day; among them, 12 had behavioral records compatible with distress, and 5 had a specific contextual factor documented. In Phase 2, descriptive reductions in EDAmax ranging from 4 to 23 µS were observed after contextual adjustments. **Conclusions**: EDA provides relevant information about autonomic arousal but should not be interpreted as a direct marker of stress. Multimodal ecological monitoring may help generate person-centered clinical hypotheses and inform individualized support in real-world settings.

## 1. Introduction

Autism spectrum disorder (ASD) is a heterogeneous neurodevelopmental condition characterized by persistent differences in social communication and interaction, together with restricted and repetitive patterns of behavior, interests, or activities [1]. At the cognitive level, autism has been associated with differences in executive functioning, central coherence, and social cognition [2,3]. Many autistic people also exhibit atypical sensory processing, expressed as hyperreactivity or hyporeactivity to auditory, visual, tactile, gustatory, olfactory, or interoceptive stimuli [4,5,6].

The DSM-5-TR describes three levels of support in ASD, with Level 3 indicating a need for very substantial support [1]. This category includes people with substantial difficulties in functional communication, behavioral regulation, environmental adaptation, and participation in everyday activities, and is frequently associated with intellectual disability. The global prevalence of autism has been estimated at approximately 1 in 100 children [7]. In the United States, a prevalence of 1 in 36 among 8-year-old children has been reported, with co-occurring intellectual disability in 37.9% of cases [8]. Nevertheless, autistic people with intellectual disability and high support needs remain underrepresented in empirical research, particularly in studies requiring self-report, experimental tasks, or laboratory participation [9].

Stress involves an organism’s response to internal or external demands and engages both the autonomic nervous system and the hypothalamic–pituitary–adrenal axis [10,11,12]. Evidence suggests that autistic people may show atypical autonomic functioning both at rest and in response to stressors [13,14]. However, autonomic functioning in autism does not follow a single profile: sympathetic hyperarousal with parasympathetic hypoarousal, hypoarousal in both systems, and other heterogeneous profiles have been described [14,15]. This variability may reflect methodological factors—including age, context, task demands, and the physiological indicators used—as well as the inherent heterogeneity of autism.

In this study, the physiological signal recorded by wearable sensors was interpreted as autonomic arousal rather than as a direct measure of distress or maladaptive stress. This distinction is essential because elevated autonomic arousal may occur in response to emotional, cognitive, sensory, social, or physical demands and does not necessarily indicate discomfort. Arousal may be consistent with an adaptive response when it occurs during a meaningful, novel, or demanding activity without behavioral signs of discomfort or functional interference. By contrast, arousal was considered compatible with distress or maladaptive stress only when it occurred alongside behavioral records of discomfort, avoidance, agitation, aggression, self-injury, freezing, or withdrawal and a documented, potentially aversive contextual factor.

Autistic people are more vulnerable to stressful and distressing experiences, which can adversely affect their physical and mental health [16,17], and have been associated with a higher risk of anxiety, depression, and suicidal ideation [18,19,20]. Among people with high support needs, identifying distress can be particularly complex because expressive communication, interoceptive awareness, and the ability to identify or verbalize internal states may be limited [21,22]. Self-report may therefore be unavailable or insufficient, and assessment should be complemented by behavioral observation, contextual information, and physiological measures.

Wearable biometric sensors allow physiological signals to be recorded noninvasively in everyday settings. Electrodermal activity (EDA) is commonly used as an indicator of sympathetic arousal because it reflects changes in skin conductance in response to internal or external stimuli [23,24]. Nevertheless, EDA should be interpreted as an indicator of autonomic arousal rather than as a specific marker of distress. Recent reviews of wearable solutions in autism highlight the potential of these technologies while also emphasizing the need for multimodal approaches, transparent signal processing, and caution when interpreting isolated physiological signals [25,26].

Ecological Momentary Assessment (EMA) provides a relevant methodological framework for investigating dynamic phenomena in daily life because it involves repeated assessments conducted in real or near-real time in natural environments, thereby reducing recall bias and increasing ecological validity [27,28]. In autistic people with high support needs, EMA-informed approaches may help characterize within-person patterns of autonomic arousal and their possible co-occurrence with behavioral and contextual records. This approach is also consistent with the small-data paradigm, which prioritizes intensive individual-level data to understand complex, dynamic, and idiosyncratic phenomena and guide personalized support [29].

Ecological Momentary Intervention (EMI) is a complementary framework referring to support or interventions delivered in daily life, in real time, and in natural settings [30]. In the present study, the second phase was treated as an exploratory case series of individualized contextual adjustments informed by EMI principles, rather than as an experimental test of effectiveness. Adjustments were implemented in everyday settings when the professional team identified a possible environmental factor associated with behavioral records compatible with distress or patterns of autonomic arousal. Given the retrospective design, the small number of cases, and the absence of an extended experimental baseline, the observed changes should be interpreted descriptively and as hypothesis-generating.

Although some studies have begun to integrate physiological signals, behavior, and context in autism [31,32,33], few have been conducted in everyday settings with autistic people who have high support needs. EMA studies in autism also indicate that emotional and biological stress reactivity do not always correspond, reinforcing the need to distinguish among the experience of stress, observable behavior, and physiological signals [34]. This gap limits the clinical applicability of findings and the development of personalized support based on ecologically valid data.

Accordingly, this study retrospectively analyzed routine-care data previously collected and stored in ABmonitor. The data were obtained in the everyday settings of autistic people with high support needs through wearable biometric sensors and professional behavioral and contextual records. The study used a retrospective, ecological, multimodal, and exploratory design and was guided by the STROBE and RECORD recommendations for observational studies and routinely collected data [35,36].

The objective was to describe patterns of autonomic arousal measured using EDA and to explore their descriptive correspondence with behavioral and contextual records compatible with distress or maladaptive stress among autistic people with high support needs in everyday settings. The research questions were: (1) How do summarized EDA values vary across participants during the monitoring period? (2) To what extent did participants with at least one day of high EDAmax also have behavioral records compatible with distress and documented contextual factors during the observed period? (3) What descriptive differences in EDAmax were observed before and after the documented contextual adjustments in the five selected cases?

## 2. Materials and Methods

### 2.1. Participants

We retrospectively analyzed secondary data from 57 people diagnosed with ASD with high support needs (Level 3 support) who attended two specialized autism services in Burgos, Spain. Data were obtained from ABmonitor, a non-public institutional database containing physiological, behavioral, and contextual information previously collected during routine care; therefore, no public repository link is available. To strengthen confidentiality, the services are referred to as Service A (*n* = 28) and Service B (*n* = 29). The mean age was 37.1 years in Service A and 18.3 years in Service B. All participants had an officially recognized disability rating greater than 65%, with an overall mean of 79% (in accordance with Spanish Royal Decree 888/2022 and medical-team assessments). Participant characteristics are presented in Table 1.

Phase 1 included all 57 participants for whom ABmonitor contained individual characteristics, routine behavioral and contextual records, and EDA summaries for at least part of the scheduled monitoring period. The schedule comprised up to 10 participant-day records per participant, distributed over approximately two calendar weeks. Participants contributed between 6 and 10 participant-day records with available EDA data. No additional signal-quality criterion or minimum-duration threshold was applied in this secondary analysis. Scheduled participant-day records without EDA data in ABmonitor were not included in the analytic dataset; any signal-quality procedures applied before storage could not be reconstructed.

Phase 2 comprised an exploratory series of five cases selected because they had: (a) available physiological, behavioral, and contextual data; (b) a specific contextual factor and behavioral records compatible with distress documented during the observed period; and (c) a documented individualized contextual adjustment implemented during routine care. The Phase 2 pre- and post-adjustment values were not obtained within the up-to-10-day monitoring period described for Phase 1. Instead, they were drawn from records collected later, at different time points during routine-care follow-up, with intervals of 28 to 91 days between the pre- and post-adjustment measurements. Adjustments included noise-canceling headphones, spatial modifications, avoidance of an aversive activity, and systematic desensitization. This phase was not interpreted as a trial, a formal EMI, an experimental study, or a test of effectiveness, but as a non-standardized exploratory description of within-case pre–post changes.

### 2.2. Instruments

Wearable biometric sensors. Empatica E4 wristbands were used to record EDA and other physiological signals [37]. The Empatica E4 has been used and validated in psychophysiological studies, although signal quality may be affected by movement, device placement, and naturalistic conditions [38,39,40,41]. For the present secondary analysis, ABmonitor provided access only to stored summary EDA data, primarily EDAmax expressed in microsiemens (µS). Raw EDA signals and synchronized accelerometry data were unavailable for reprocessing, artifact detection, or segment-level quality assessment. EDA was conceptualized as an indicator of autonomic arousal rather than as a direct or specific measure of distress [23,24].

Mobile application. Professionals used the organization’s EDATEA mobile application to record behavioral and contextual variables systematically and in real or near-real time. Behavioral records included the activity, observable behavior, possible discomfort, avoidance, agitation, aggression, self-injury, freezing, withdrawal, and other relevant manifestations.

Web application. The organization’s ABmonitor web application integrated physiological, behavioral, and contextual data into a single database. It also supported data visualization, traceability, and the extraction of additional variables, including sex, age, sensory disability, history of epilepsy, and disability percentage.

### 2.3. Procedure

This retrospective, descriptive secondary analysis used ecological data previously collected during routine care and stored in ABmonitor. It comprised two related analytic phases (Table 2).

The secondary-analysis protocol was approved by the Institutional Research Ethics Committee of Universidad Continental, Peru (approval letter No. 0674-2023-CIEI-UC, 30 October 2023). The research team did not recontact participants or access direct identifiers. Legal representatives or caregivers had provided informed consent for the original data collection.

Professionals responsible for routine behavioral recording received six hours of in-person training, delivered in two three-hour sessions by specialists from the research team. Training covered the recognition of observable behaviors, use of the mobile application, identification of contextual factors, and practical recording exercises. No standardized post-training competency assessment, calibration sessions, or estimates of interobserver agreement were documented; this was considered a limitation.

Potential adverse reactions to the device were monitored. Initially, two participants showed discomfort, expressed through withdrawal or rejection of the wristband. Gradual desensitization strategies, including brief sessions with positive reinforcement, were used in these cases. No participant was excluded for this reason.

EDA was used as the primary physiological variable of autonomic arousal. The central descriptive variable was daily maximum EDA (EDAmax), expressed in µS. Three internal operational arousal categories were retained: low (EDAmax < 1 µS), medium (1 µS ≤ EDAmax < 10 µS), and high (EDAmax ≥ 10 µS). These cutoffs are presented as internal descriptive criteria for the project rather than as universal stress thresholds. When exportable data permit, interpretation should be supplemented with within-person indicators such as the daily median, 95th percentile, peak frequency, duration of arousal episodes, and change relative to the individual’s baseline pattern.

The analysis used daily EDA summaries stored in ABmonitor rather than raw physiological signals. The monitoring schedule comprised 570 expected participant-day records (57 participants with up to 10 scheduled days). ABmonitor contained EDA data for 532 participant-day records; the remaining 38 expected records did not contain EDA data and were excluded from the analysis. This secondary analysis did not apply additional duration thresholds, segment removal, raw-signal filtering, artifact correction, accelerometry-assisted detection, or signal resynchronization. The original procedures used to detect signal loss, manage movement artifacts, identify extreme values, and exclude segments could not be reconstructed from the available documentation. Consequently, the original physiological preprocessing cannot be reproduced.

In parallel with EDA monitoring, professionals used EDATEA to record routine behavioral and contextual information, including the activity, observable behavior, perceived discomfort or distress, and potentially relevant stimuli or conditions such as noise, temperature changes, auditory, visual, gustatory, olfactory, or tactile stimuli, community outings, and activity transitions. When available, timestamps from professional records informed the joint review. However, no uniform time window, reproducible episode-to-signal matching algorithm, or validated classification rule was applied. Contextual factors are therefore reported as conditions documented during the observed period, not as demonstrated causes or episode-level correlates.

### 2.4. Data Analysis

Quantitative summaries were combined with a structured descriptive review of routine behavioral and contextual records in ABmonitor. The domains reviewed—activity, observable behavior, perceived distress, and contextual factors—were derived from pre-existing system fields and notes. Categorical variables were summarized using frequencies and percentages. Continuous variables were described using means and standard deviations or medians and interquartile ranges, as appropriate. No inferential, correlational, or time-series analyses were conducted. Analyses were performed in Stata 17 (StataCorp LLC, College Station, TX, USA).

The analytic workflow comprised: (1) extraction from ABmonitor of participant characteristics, available daily EDA summaries, and professional behavioral and contextual records; (2) identification of the 570 scheduled participant-day records and inclusion of the 532 records containing EDA data; (3) description of EDAmax values and categories across participant-day records, participants, and services; (4) structured participant-level review of co-occurrence during the observed period among at least one day of high EDAmax, behavioral records compatible with distress, and documented contextual factors; and (5) non-standardized descriptive pre–post comparison of EDAmax in the five selected cases.

To address the first research question, variation in EDA was described across participants, considering available days and services. For the second question, descriptive co-occurrence of physiological, behavioral, and contextual information was examined at the participant level. For the third question, absolute differences and changes in EDAmax category before and after the documented contextual adjustments were calculated.

Potential sources of bias included participant selection, professional observation and recording, contextual confounding, regression to the mean, and variability related to age, service, activity, and clinical characteristics. A particularly important limitation was that the analysis relied exclusively on EDA summaries stored in ABmonitor, without access to raw physiological signals or complete documentation of preprocessing. This prevented independent assessment of signal quality, identification of movement artifacts and extreme values, quantification of data loss, or verification of the original segment-exclusion criteria, thereby introducing a substantial risk of measurement bias. In addition, purposive selection of the five Phase 2 cases may have favored cases with more complete information or more evident patterns. These sources of bias were considered when interpreting the findings and are explicitly addressed in the study limitations.

## 3. Results

### 3.1. EDA Patterns in Everyday Settings

During Phase 1, ABmonitor contained EDA data for 532 of the 570 participant-day records specified in the monitoring schedule (93.3%). The remaining 38 expected participant-day records (6.7%) did not contain EDA data and were excluded from the analysis. Participants contributed between 6 and 10 participant-day records with available EDA data, and 37 of the 57 participants had data for all 10 scheduled days. The available records comprised 2216 h and 37 min. Among participant-day records with available EDA data, the median recording duration was 260 min (interquartile range: 20); the 38 expected participant-day records without EDA data were not included in this duration calculation.

Daily maximum EDA (EDAmax) was examined for each participant. When the records were categorized, 11 participants (19.3%) consistently remained in the same autonomic arousal category across all study days: 6 low, 4 medium, and 1 high. The remaining participants (80.7%) had variable records, with low, medium, or high arousal on different days (Table 3). These findings indicate substantial within- and between-person variability in autonomic arousal and support the need for person-centered analyses. Elevated EDA was not interpreted in isolation as distress, but as autonomic arousal requiring multimodal interpretation.

### 3.2. Descriptive Co-Occurrence of EDA, Behavior, and Context

No direct or one-to-one relationship was observed between elevated EDA values and records compatible with distress. For example, one participant showed elevated arousal during a music workshop without behavioral signs of discomfort, suggesting autonomic arousal that was not necessarily maladaptive. Conversely, another participant showed avoidance and anticipatory behavior during a community outing while EDA was minimal, suggesting possible discordance among observable behavior, inferred internal experience, and the recorded physiological response.

Overall, 22 of the 57 participants (38.6%) showed high autonomic arousal on at least one day. Of these 22 participants, 12 showed co-occurrence during the observed period between elevated arousal and behavioral records compatible with distress, such as avoidance, agitation, or aggressive behavior, as well as professional reports of discomfort. This classification was interpreted as exploratory and multimodal, rather than as an objective identification of distress based solely on EDA.

In five cases, a specific contextual factor and behavioral records compatible with distress were documented during the observed period. These factors included noise or crowding in external environments, occasional encounters with dogs, and participation in gym activities. The factors are described as contextual conditions documented during the observed period rather than as demonstrated causes or episode-level correlates.

### 3.3. Descriptive Changes After Individualized Contextual Adjustments

Phase 2 comprised five selected cases with a documented contextual adjustment and EDAmax values available before and after the adjustment. These measurements were obtained after the Phase 1 monitoring period, with intervals of 28 to 91 days between the pre- and post-adjustment measurements. Post-adjustment EDAmax values were 4 to 23 µS lower than the corresponding pre-adjustment values across these five records. The three cases initially categorized as high shifted to the medium category, and the two initially categorized as medium shifted to the low category (Table 4). These comparisons were not standardized, and neither the total number of contextual adjustments in the source project nor the number of cases without lower post-adjustment values was available.

Adjustments were individualized according to the contextual factor identified. Systematic desensitization was used in two cases involving occasional encounters with dogs. In one case involving gym exercises, the activity was replaced by an alternative functional activity in a different space. In two cases involving noise or crowding in external environments, noise-canceling headphones were provided during community outings. The observed changes were compatible with reduced autonomic arousal; however, because standardized pre–post behavioral data were unavailable, no conclusion can be drawn regarding behavioral change, causality, or intervention effectiveness.

## 4. Discussion

This study described variability in the EDA summaries stored in ABmonitor and explored their participant-level co-occurrence with routine behavioral and contextual records during the observed period among autistic people with high support needs. It also reported non-standardized pre–post differences in EDAmax in five selected cases with documented contextual adjustments.

Phase 1 showed high within- and between-person variability in EDA. This finding is consistent with reviews of autonomic functioning in autism, which report heterogeneous profiles and non-convergent results across studies [13,14]. It is also consistent with the literature suggesting that physiological responses in autism depend on context, task demands, age, clinical characteristics, and the indicators used [14]. The contribution of the present study lies in demonstrating this variability using ecological data from people with high support needs, a population often underrepresented in research.

The findings also showed that elevated autonomic arousal did not necessarily coincide with observable distress. In some cases, high EDA occurred during activities without behavioral signs of discomfort, whereas in others, avoidance behavior occurred alongside low EDA. This discordance is consistent with studies describing differences among physiological arousal, emotional expression or communication, and the subjective experience of autistic people [22,42]. Van Der Linden et al. similarly distinguished emotional from biological stress reactivity in daily life and showed that these dimensions do not always behave equivalently [34]. Interpreting EDA as stress without contextual and behavioral information may therefore lead to misleading conclusions.

The value of an EMA framework lies in its capacity to capture everyday variability rather than being limited to responses during brief laboratory tasks. Previous work has demonstrated the relevance of momentary and naturalistic assessment for understanding stress and arousal in autism [28,34]. Unlike studies based on brief experimental tasks, the present study analyzed repeated records collected over two weeks in everyday settings. This increases ecological validity but reduces experimental control and requires cautious interpretation.

Phase 2 yielded clinically suggestive but methodologically preliminary findings. In the five cases with a documented contextual factor, individualized adjustments were descriptively associated with reductions in EDA. This pattern is consistent with small-data and precision-oriented approaches, which prioritize within-person understanding and personalized support [29,43]. However, owing to the retrospective design, small number of cases, absence of an extended experimental baseline, and variable intervals between measurements, these findings do not demonstrate effectiveness or causality. Instead, they generate hypotheses regarding the potential value of individualized environmental adjustments.

From a digital-health perspective, the findings are consistent with recent reviews highlighting the potential of wearable devices in autism while emphasizing the need for multimodal analyses, transparent signal processing, and validation in real-world settings [25,26]. Studies using wearable biosensors have contributed to the detection or prediction of agitation and aggressive behavior in autism [44,45], but current evidence continues to support caution when translating physiological signals into clinical interpretations. Future research should integrate heart rate, heart rate variability, temperature, accelerometry, synchronized contextual records, and, when possible, subjective or proxy-reported measures.

This study has several limitations. First, the analysis relied exclusively on EDA data stored in ABmonitor, without access to raw physiological signals or complete documentation of their preprocessing. It was therefore not possible to independently verify signal quality, identify movement artifacts or extreme values, quantify data loss, or reproduce the original segment-exclusion criteria; this represents an important source of measurement bias. In addition, EDA was the only physiological signal analyzed, and no other autonomic, endocrine, or movement measures were integrated to support a more robust multimodal interpretation. Second, behavioral and contextual information was recorded by professionals and was therefore proxy-reported. Although the professionals received training, no standardized competency assessments, calibration sessions, or measures of interobserver agreement were available, limiting the reliability and comparability of the records. Third, the retrospective design based on routine-care data is susceptible to selection, recording, observation, and information biases, as well as contextual confounding. Fourth, purposive selection of the five Phase 2 cases may have favored cases with more complete information or more evident patterns. This phase also lacked an extended experimental baseline, random assignment, standardized pre–post behavioral measures, and systematic control of external variables; the observed changes therefore cannot be causally attributed to the contextual adjustments or interpreted as evidence of effectiveness. Finally, heterogeneity in age, clinical characteristics, activities, and institutional contexts may have influenced the observed patterns and limits the generalizability of the findings.

The main strengths of this study are the inclusion of autistic people with high support needs, the use of everyday data from real-world settings, the extended duration of physiological recording, and the integration of physiological, behavioral, and contextual information. Future studies should use prospective designs with prespecified signal-processing protocols, criteria for valid records, interobserver reliability, multimodal measures, and within-person indicators. Single-case experimental designs, multiple-baseline designs, and intensive time-series methods may be particularly useful for evaluating contextual adjustments with greater methodological rigor.

## 5. Conclusions

This study shows that autistic people with high support needs exhibit heterogeneous patterns of autonomic arousal in everyday settings. EDA provides relevant information about autonomic arousal but cannot, on its own, identify maladaptive stress. Its interpretation must be integrated with behavioral and contextual records, particularly when self-report is unavailable. Phase 1, informed by retrospective EMA principles, made it possible to describe within- and between-person variability. Phase 2, conceived as an exploratory case series informed by EMI principles, showed descriptive changes after individualized contextual adjustments but did not permit causal inference. These findings support the value of multimodal ecological monitoring and small-data approaches for generating clinical hypotheses and informing personalized support in real-world settings.

## Figures and Tables

**Table 1:** Participant characteristics in Phase 1.

Characteristic	Service A (*n* = 28)	Service B (*n* = 29)
Male, *n* (%)	19 (67.9)	26 (89.7)
Female, *n* (%)	9 (32.1)	3 (10.3)
Age ≥ 18 years, *n* (%)	28 (100.0)	8 (27.6)
Age, years, mean (SD)	37.1 (9.7)	18.3 (7.6)
Sensory disability, *n* (%)	0 (0.0)	5 (17.2)
Epilepsy, *n* (%)	7 (25.0)	5 (17.2)
Estimated disability percentage, mean (SD)	81.1 (7.5)	77.8 (8.7)
Median of daily median EDA, µS, median (IQR)	2.5 (7.3)	2.3 (5.2)
At least one day with medium EDAmax, *n* (%)	25 (89.2)	26 (89.6)
At least one day with high EDAmax, *n* (%)	13 (46.4)	9 (31.0)
Daily recording duration among participant-days with available EDA data, min, median (IQR)	260.0 (18.0)	259.0 (29.0)
Same EDAmax category across days, *n* (%)	5 (17.9)	6 (20.7)

**Table 2:** Retrospective ecological design and analytic phases. EDA = electrodermal activity.

Phase 1: Repeated Ecological Monitoring	Multimodal Interpretation	Phase 2: Selected Descriptive Comparisons
• 57 autistic people with high support needs. • Up to 10 scheduled participant-day records; 6–10 with available EDA data per participant. • EDA summaries obtained using a wearable device and behavioral and contextual records stored in ABmonitor.	• Joint interpretation of physiological, behavioral, and contextual information. • Exploration of their co-occurrence during the observed period. • Focus on individual-level patterns.	• Five cases with documented, individualized contextual adjustments. • Non-standardized pre–post comparisons of EDAmax. • No standardized pre–post behavioral measure or inference of effectiveness or causality.

**Table 3:** Summary of EDA patterns and multimodal interpretation in Phase 1.

Indicator	Value
Total participants	57
Participant-day records with EDA data	532
Total recording time	2216 h 37 min
Daily recording time per participant, median (IQR)	260 min (20.0)
Consistent arousal category across days	11/57 (19.3%)
Consistently low arousal	6/57
Consistently medium arousal	4/57
Consistently high arousal	1/57
Variable arousal category across days	46/57 (80.7%)
High autonomic arousal on at least one day	22/57 (38.6%)
High arousal with behavioral records compatible with distress	12/22
Specific contextual factor documented	5/57

Note: The categories are internal descriptive criteria and should not be interpreted as universal stress thresholds.

**Table 4:** Exploratory case series of contextual adjustments and descriptive pre–post changes in EDAmax during Phase 2.

Code	Context/Behavior	Contextual Adjustment	Interval	Pre-Adjustment EDAmax	Post-Adjustment EDAmax (Δ)
CDB18	Gym activities; progressive nervousness during the activity	The gym activity was discontinued and replaced with an alternative document-shredding workshop	91 days	25.08 µS (high)	2.06 µS (medium); Δ = −23.02 µS
EAB13	Occasional encounters with dogs	Systematic desensitization	49 days	18.12 µS (high)	7.53 µS (medium); Δ = −10.59 µS
EAB14	Occasional encounters with dogs	Systematic desensitization	49 days	9.69 µS (medium)	0.49 µS (low); Δ = −9.20 µS
EAB15	Noise or crowding in external settings	Noise-canceling headphones during community outings	28 days	23.72 µS (high)	8.73 µS (medium); Δ = −14.99 µS
EAB16	Noise or crowding in external settings	Noise-canceling headphones during community outings	35 days	4.57 µS (medium)	0.57 µS (low); Δ = −4.00 µS

Note: Low, medium, and high are internal descriptive categories based on EDAmax. The observed changes are descriptive and do not establish causal effectiveness.

## Data Availability

The data supporting the findings of this study are not publicly available because of privacy, confidentiality, and ethical restrictions relating to a vulnerable population. Deidentified data may be requested from the corresponding author upon reasonable request, subject to approval by the relevant institutions and ethics committee, and only when compatible with the terms of consent and data-protection requirements.

## Abbreviations

The following abbreviations are used in this manuscript:

ANS	autonomic nervous system
ASD	autism spectrum disorder
EDA	electrodermal activity
EMA	ecological momentary assessment
EMI	ecological momentary intervention
µS	microsiemens
